# Supplementary material for: A pair of transporters controls mitochondrial Zn2+ levels to maintain mitochondrial homeostasis
Source: Protein Cell. 2021 Oct 23;13(3):180–202. doi: 10.1007/s13238-021-00881-4 (PMC8901913; doi:10.1007/s13238-021-00881-4)
Supplement: Supplementary file 1 — Supplementary file1 (PDF 154177 kb) [file 13238_2021_881_MOESM1_ESM.pdf]

Supplemental materials

Figure S1. Characterization of the mitochondrial defects in *slc-30A9(yq158)* mutants.

(**A**) Images of mitochondria labeled with F54A3.5::GFP and TOMM-20::mCh in the hypodermis of N2 and *yq158* animals. Bars, 5 μm.

(**B**) Representative TEM images of mitochondria in the germline, sperm, oocyte and embryo of N2 and *yq158* animals. Bars, 1 μm.

(**C**) Time-course comparison of Mito-GFP-labeled mitochondria in the hypodermis of N2 and *yq158* animals. Bars, 5 μm.

(**D**) Left: Representative images of mitochondria in the hypodermis of control RNAi-and *eat-3* RNAi-treated N2 and *yq158* animals. Right: Images of mitochondria in the hypodermis of N2, *yq158*, *drp-1(tm1108),* and *yq158;drp-1(tm1108)* mutants. Bars, 5 μm.

(**E**) Expression of $P_{slc\text{-}30A9}$*slc-30A9*::*GFP* in hypodermal, muscle and intestinal cells as well as in the PVD neuron in N2 adults. DIC and fluorescence images are shown for each tissue. Bars, 5 μm.

Figure S2. Amino acid sequence alignment of bacterial (*E.c*) Yiip, *C. elegans* SLC-30A9 and human (*H.s*) SLC30A9. Residues important for Zn^{2+} binding are indicated in red boxes. Mitochondrion-targeting sequences (MTSs) are indicated in blue boxes. The cation-efflux domains are indicated in pink boxes. Residues important for Zn^{2+} binding in Yiip and SLC-30A9 are labeled in blue and red, respectively.

Figure S3. Characterization of SLC30A9 localization and binding with divalent cations.

(**A**) Localization of *C. elegans* SLC-30A9-EGFP, SLC-30A9(1-145)-EGFP, and SLC-30A9(146-495)-EGFP in HeLa cells. Mitochondria are labeled with MitoTracker DR. Boxed regions are magnified (2.5×) in the insets. Bars, 2 μm.

(**B**) Binding curves of HEK293 cell-expressed SLC-30A9-EGFP with divalent cations (Ca^{2+}, Mg^{2+}, Cu^{2+} and Mn^{2+}), measured with MST assays. EGFP was used as the negative control.

(**C**) Co-localization of human SLC30A9-EGFP with MitoTracker DR in HeLa cells. Boxed regions are magnified (2×) in the insets. Bars, 2 μm.

(**D**) Binding curve of HEK293 cell-expressed human SLC30A9-EGFP with Zn^{2+}, measured with MST assays. EGFP was used as the negative control.

Figure S4. Characterization of *C. elegans* SLC-25A25.

(A) Amino acid sequence alignment of *C. elegans* (*C. e*) SLC-25A25 and human (*H. s*) SLC25A25. EF-hands are indicated in red boxes. Mitochondrial carrier domains are indicated in blue boxes.

(B) Expression pattern of $P_{slc\text{-}25A25}$*slc-25A25*::*GFP* in *C. elegans*. DIC and fluorescence images are shown for the indicated tissues. Bars, 5 μm.

(**C**) Representative images of mitochondria in the hypodermis of N2 and *slc-25A25*(*yq406*) animals treated with control RNAi, *drp-1* RNAi, and *eat-3* RNAi.

Bars, 5 µm.

Figure S1

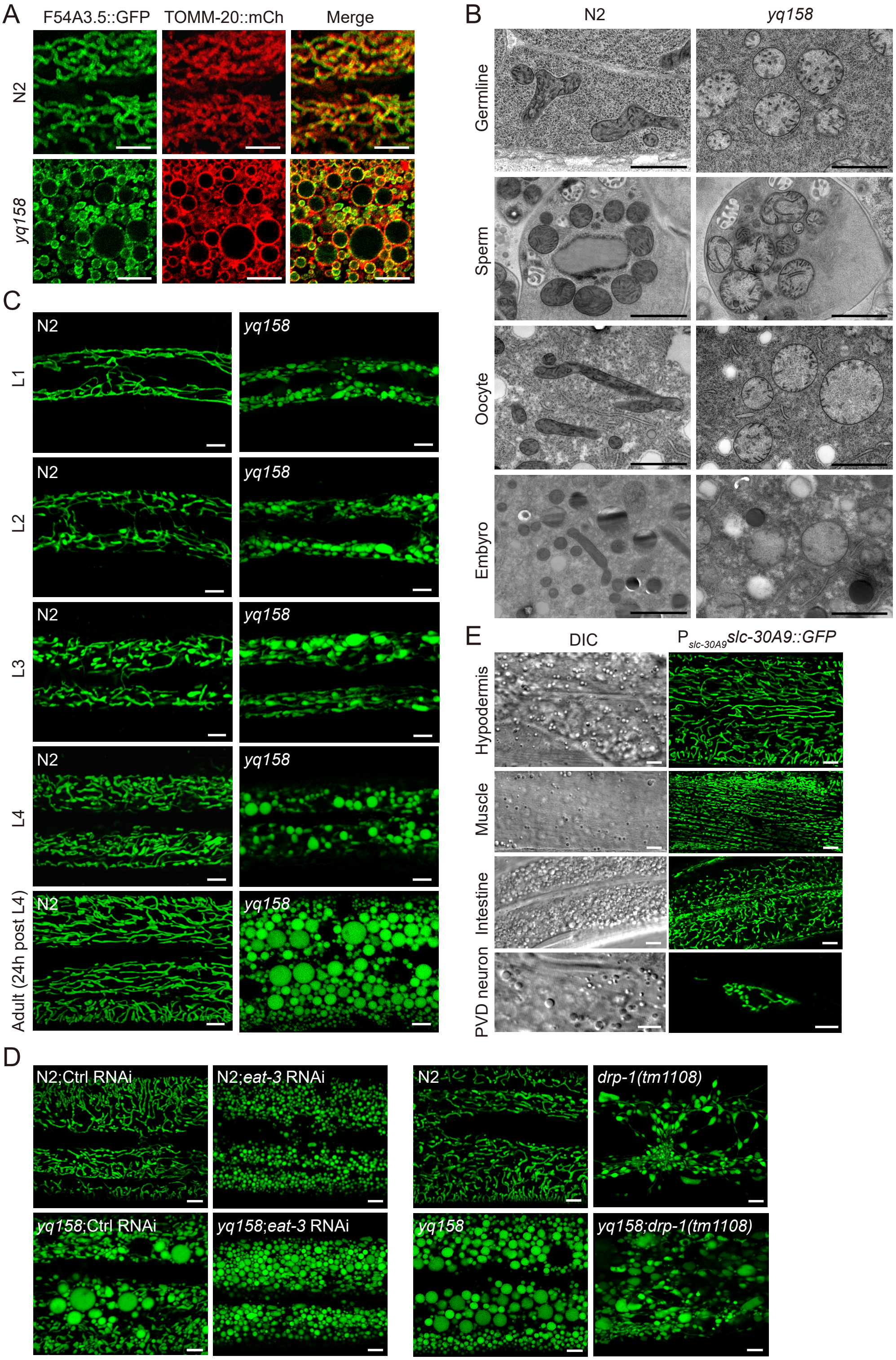
A
F54A3.5::GFP
TOMM-20::mCh
Merge
N2
yq158
B
N2
yq158
Germline
Sperm
Oocyte
Embyro
C
L1
L2
L3
L4
Adult (24h post L4)
N2
yq158
E
DIC
P slc-30A9 slc-30A9::GFP
Hypodermis
Muscle
Intestine
PVD neuron
D
N2;Ctrl RNAi
N2;eat-3 RNAi
N2
drp-1(tm1108)
yq158;Ctrl RNAi
yq158;eat-3 RNAi
yq158
yq158;drp-1(tm1108)

Figure S2

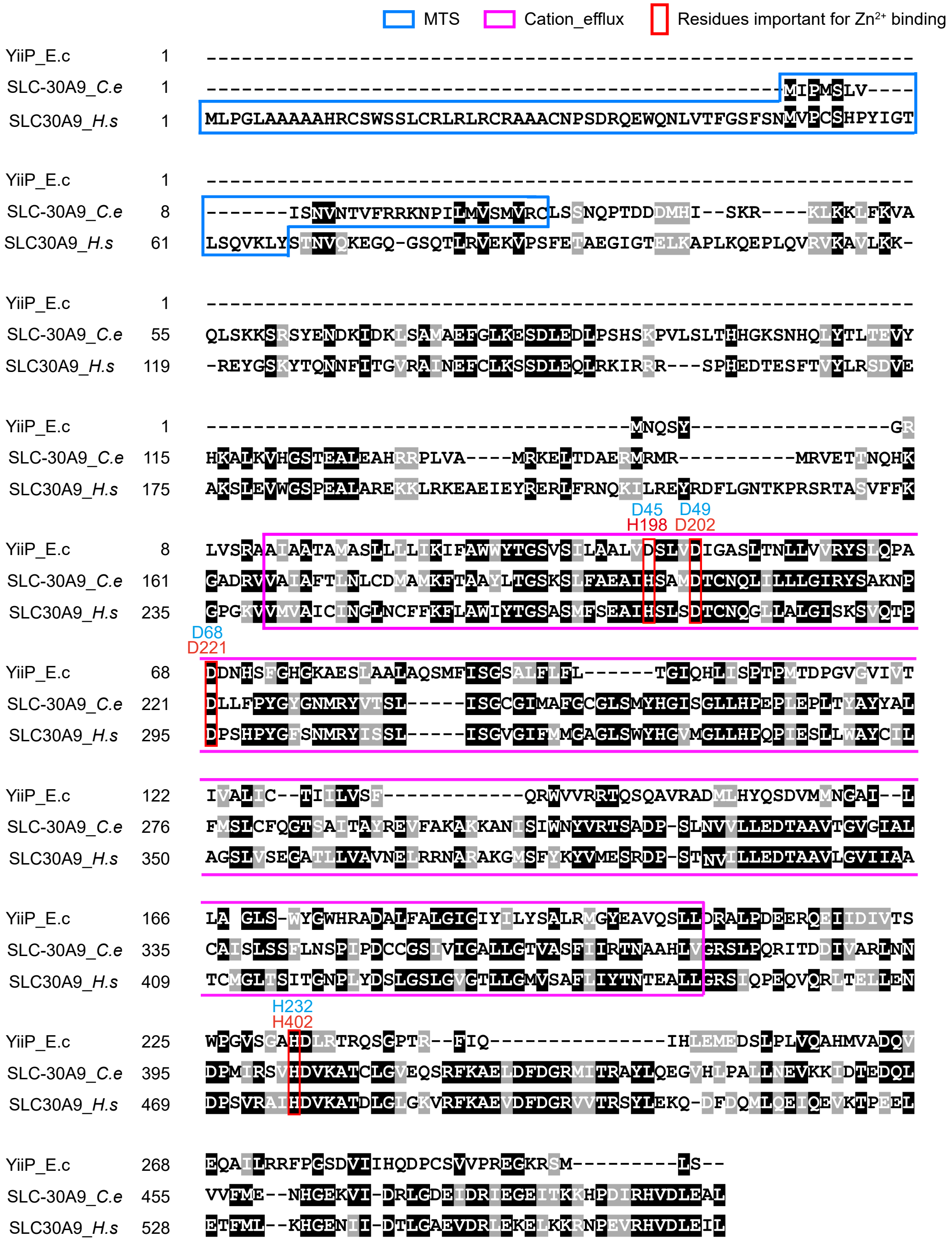
MTS
Cation_efflux
Residues important for Zn^{2+} binding
YiiP_E.c 1 --
SLC-30A9_C.e 1 --MIPMSLV----
SLC30A9_H.s 1 MLPGLAAAAAHRCSWSSLCRLRLRCRAAACNPSDRQEWQNLVTFGSFSNMVPCSHPYIGT
YiiP_E.c 1 --
SLC-30A9_C.e 8 -------ISNVNTVFRRKNPILMVSMVRCLSSNQPTDDDMHI--SKR----KLKKLFKVA
SLC30A9_H.s 61 LSQVKLYSTNVQKEGQ-GSQTLRVEKVPSFETAEGIGTELKAPLKQEPLQVRVKAVLKK-
YiiP_E.c 1 --
SLC-30A9_C.e 55 QLSKKSRSYENDKIDKLSAMAEFGLKESDLEDLPSHSKPVLSLTHHGKSNHQLYTLTEVY
SLC30A9_H.s 119 -REYGSKYTQNNFITGVRAINEFCLKSSDLEQLRKIRR---SPHEDTESFTVYLRSDVE
YiiP_E.c 1 -------------------------------------MNQSY-----------------GR
SLC-30A9_C.e 115 HKALKVHGSTEALEAHRRPLVA----MRKELTDAERMRMR----------MRVETTNQHK
SLC30A9_H.s 175 AKSLEVWGSPEALAREKKLRKEAEIEYRERLFRNQKILREYRDFLGNTKPRSRTASVFFK
D45 D49
H198 D202
YiiP_E.c 8 LVSRAAIAATAMASLLLLIKIFAWWYTGSVSILAALVDSLVDIGASLTNLLVVRYSLQPA
SLC-30A9_C.e 161 GADRVVAIAFTLNLCDMAMKFTAAYLTGSKSLFAEAIHSAMDTCNQLILLLGIRYSAKNP
SLC30A9_H.s 235 GPGKVVMVAICINGLNCFFKFLAWIYTGSASMFSEAIHSLSDTCNQGLLALGISKSVQTP
D68
D221
YiiP_E.c 68 DDNHSFGHGKAESLAALAQSMFISGSALFLFL------TGIQHLISPTPMTDPGVGVIVT
SLC-30A9_C.e 221 DLLFPYGYGNMRYVTSL-----ISGCGIMAFGCGLSMYHGISGLLHPEPLEPLTYAYYAL
SLC30A9_H.s 295 DPSHPYGFSNMRYISSL-----ISGVGIFMMGAGLSWYHGVMGLLHPQPIESLLWAYCIL
YiiP_E.c 122 IVALIC--TIILVSF------------QRWVVRRTQSQAVRADMLHYQSDVMMNGAI--L
SLC-30A9_C.e 276 FMSLCFQGTSAITAYREVFAKAKKANISIWNYVRTSADP-SLNVVLLEDTAAVTGVGIAL
SLC30A9_H.s 350 AGSLVSEGATILVAVNELRRNARAKGMSFYKYVMESRDP-STNVILLEDTAAVLGVIIAA
YiiP_E.c 166 LA GLS-WYGWHRADALFALGIGIYILYSALRMGYEAVQSLLDRALPDEERQEIIDIVTS
SLC-30A9_C.e 335 CAISLSSFLNSPIPDCCGSIVIGALLGTVASFIIRTNAAHLVGRSLPQRITDDIVARLNN
SLC30A9_H.s 409 TCMGLTSITGNPLYDSLGSLGVGTLLGMVSAFLIYTNTEALLGRSIQPEQVQRLTELLEN
H232
H402
YiiP_E.c 225 WPGVSGAHDLRTRQSGPTR--FIQ--------------IHLEMEDSLPLVQAHMVADQV
SLC-30A9_C.e 395 DPMIRSVHDVKATCLGVEQSRFKAELDFDGRMITRAYLQEGVHLPALLNEVKKIDTEDQL
SLC30A9_H.s 469 DPSVRAIHDVKATDLGLGKVRFKAEVDFDGRVVTRSYLEKQ-DFDQMLQEIQEVKTPEEL
YiiP_E.c 268 EQAIIRRFPGSDVIIHQDPCSVVPREGKRSM---------LS--
SLC-30A9_C.e 455 VVFME--NHGEKVI-DRLGDEIDRIEGEITKKHPDIRHVDLEAL
SLC30A9_H.s 528 ETFML--KHGENII-DTLGAEVDRLEKELKKRNPEVRHVDLEIL

Figure S3

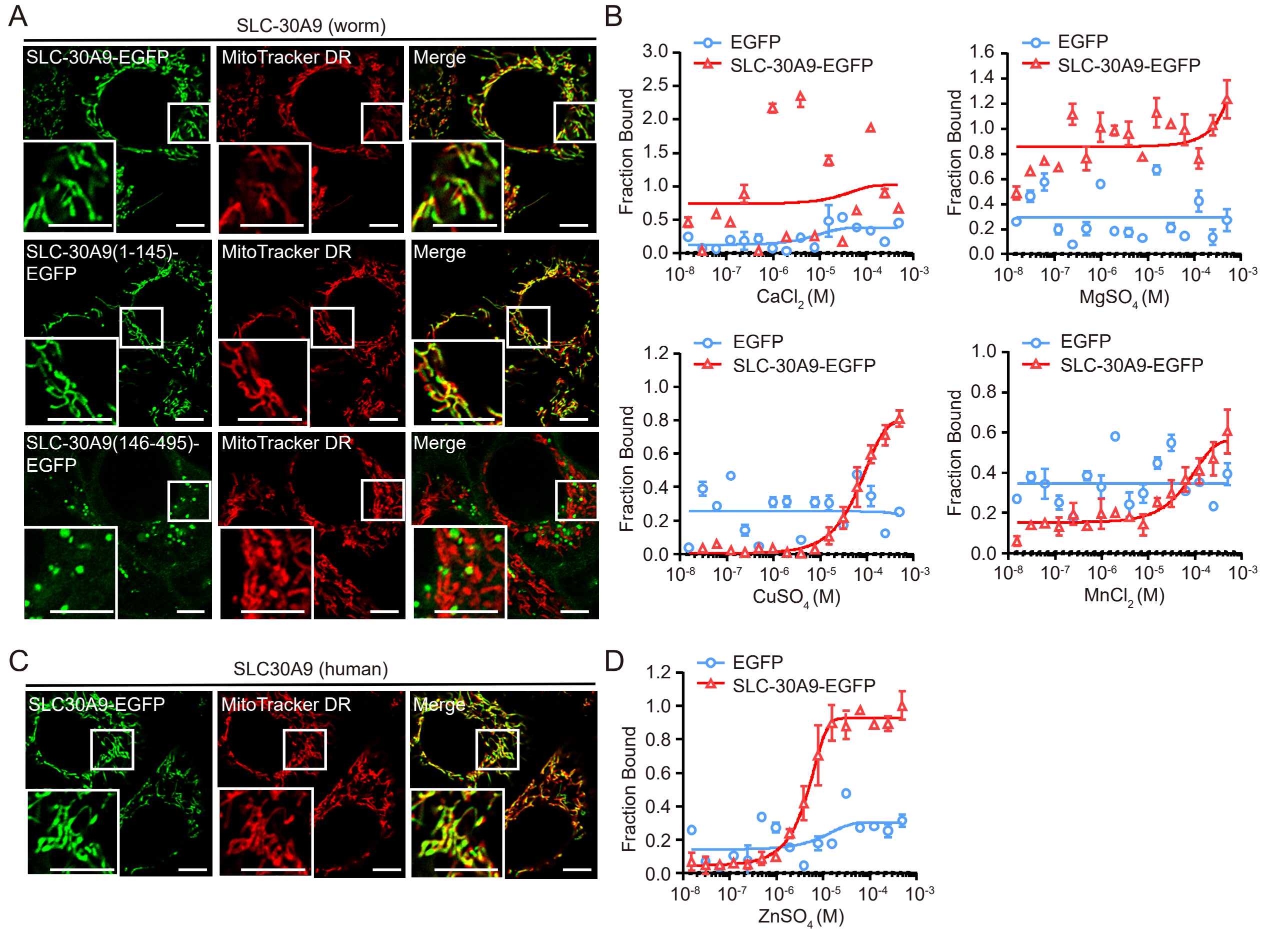
A
SLC-30A9 (worm)
SLC-30A9-EGFP
MitoTracker DR
Merge
SLC-30A9(1-145)-EGFP
SLC-30A9(146-495)-EGFP
B
EGFP
SLC-30A9-EGFP
Fraction Bound
CaCl2 (M)
MgSO4 (M)
CuSO4 (M)
MnCl2 (M)
C
SLC30A9 (human)
SLC30A9-EGFP
MitoTracker DR
Merge
D
EGFP
SLC-30A9-EGFP
Fraction Bound
ZnSO4 (M)

Figure S4

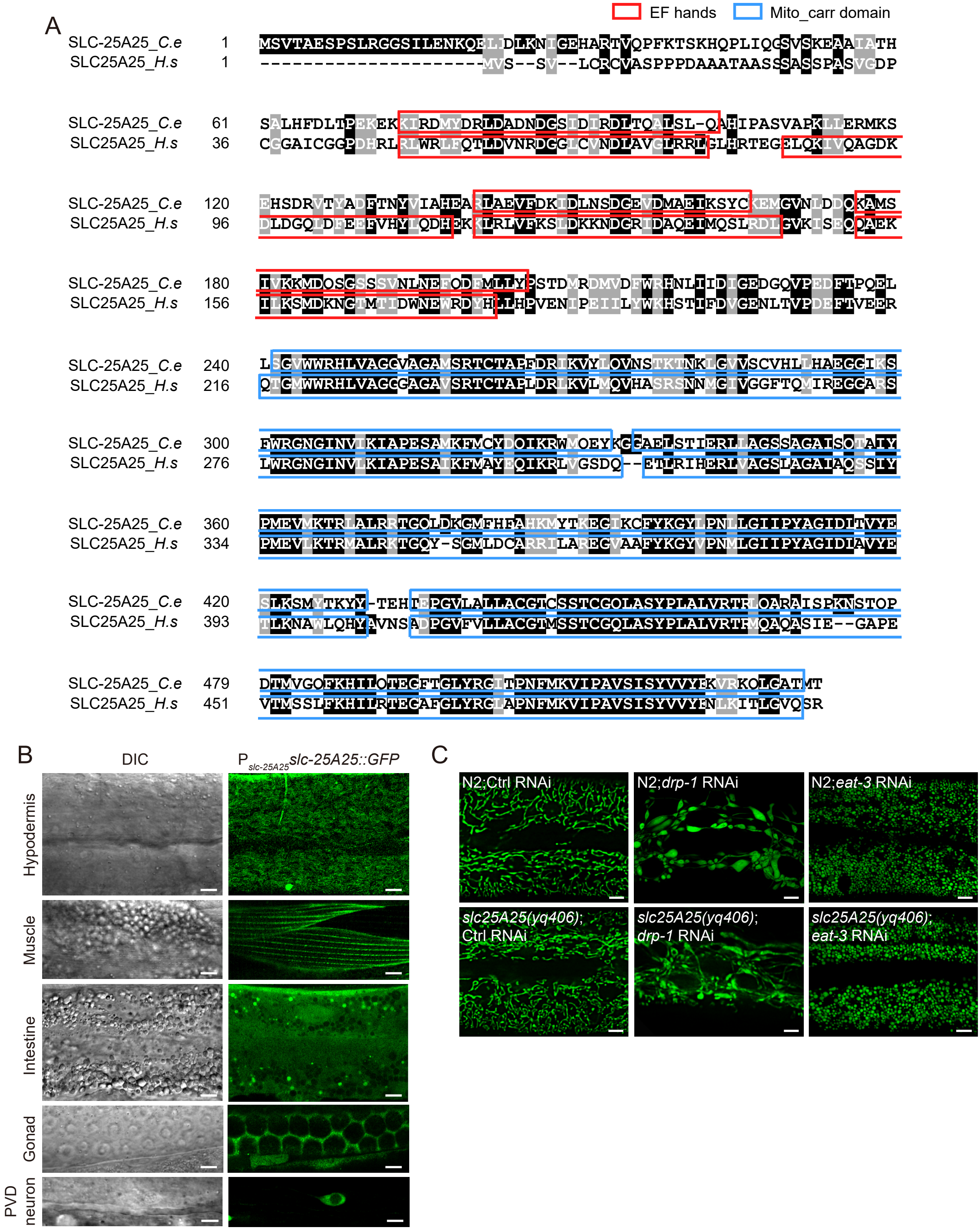

EF hands
Mito_carr domain
A
SLC-25A25_C.e 1 MSVTAESPSLRGGSILENKQELLDLKNIGEHARTVQPFKTSKHQPLIQGSVSKEAAIATH
SLC25A25_H.s 1 ---------------------MVS--SV--LCRCVASPPPDAAATAASSSASSPASVGDP
SLC-25A25_C.e 61 SALHFDLTPEKEKKIRDMYDRLDADNDGSIDIRDLTQALSL-QAHIPASVAPKLLERMKS
SLC25A25_H.s 36 CGGAICGGPDHRLRLWRLFQTLDVNRDGGICVNDLAVGLRRLGLHRTEGELQKIVQAGDK
SLC-25A25_C.e 120 EHSDRVTYADFTNYVIAHEARLAEVFDKIDLNSDGEVDMAEIKSYCKEMGVNLDDQKAMS
SLC25A25_H.s 96 DLDGQLDEEEFVHYLQDHEKKLRLVFKSLDKKNDGRIDAQEIMQSLRDLGVKISEQQAEK
SLC-25A25_C.e 180 IVKKMDQSGSSSVNLNEFQDFMLLYPSTDMRDMVDFWRHNLIIDIGEDGQVPEDFTPQEL
SLC25A25_H.s 156 IIKSMDKNGTMTIDWNEWRDYHLLHPVENIPEIILYWKHSTIFDVGENLTVPDEFTVEER
SLC-25A25_C.e 240 LSGVWWRHLVAGGVAGAMSRTCTAPFDRIKVYLQVNSTKTNKLGVVSCVHLLHAEGGIKS
SLC25A25_H.s 216 QTGMWWRHLVAGGGAGAVSRTCTAPLDRLKVLMQVHASRSNNMGIVGGFTQMIREGGARS
SLC-25A25_C.e 300 FWRGNGINVIKIAPESAMKFMCYDQIKRWMQEYKGGAELSTIERLLAGSSAGAISQTAIY
SLC25A25_H.s 276 LWRGNGINVLKIAPESAIKFMAYEQIKRLVGSDQ--ETLRIHERLVAGSLAGAIAQSSIY
SLC-25A25_C.e 360 PMEVMKTRLALRRTGQLDKGMFHFAHKMYTKEGIKCFYKGYLPNLLGIIPYAGIDLTVYE
SLC25A25_H.s 334 PMEVLKTRMALRKTGQY-SGMLDCARRILAREGVAAFYKGYVPNMLGIIPYAGIDIAVYE
SLC-25A25_C.e 420 SLKSMYTKYY-TEHTEPGVLALLACGTCSSTCGQLASYPLALVRTRLQARAISPKNSTQP
SLC25A25_H.s 393 TLKNAWLQHYAVNSADPGVFVLLACGTMSSTCGQLASYPLALVRTRMQAQASIE--GAPE
SLC-25A25_C.e 479 DTMVGQFKHILQTEGFTGLYRGITPNFMKVIPAVSISYVVYEKVRKQLGATMT
SLC25A25_H.s 451 VTMSSLFKHILRTEGAFGLYRGLAPNFMKVIPAVSISYVVYENLKITLGVQSR
B
DIC
P slc-25A25 slc-25A25::GFP
Hypodermis
Muscle
Intestine
Gonad
PVD neuron
C
N2;Ctrl RNAi
N2;drp-1 RNAi
N2;eat-3 RNAi
slc25A25(yq406); Ctrl RNAi
slc25A25(yq406); drp-1 RNAi
slc25A25(yq406); eat-3 RNAi
